# Gestational diabetes perception profiles based on attachment style: a cross-sectional study

**DOI:** 10.1007/s00592-024-02251-y

**Published:** 2024-03-13

**Authors:** Ana Munda, Katarina Lia Kompan Erzar, Helena Peric, Draženka Pongrac Barlovič

**Affiliations:** 1 Department of Endocrinology, Diabetes, and Metabolic Diseases, University Medical Centre Ljubljana, Zaloska Cesta 7, 1000 Ljubljana, Slovenia; 2 Faculty of Medicine, University of Ljubljana, Ljubljana, Slovenia; 3 Faculty of Theology, University of Ljubljana, Ljubljana, Slovenia; 4 Faculty of Arts, University of Ljubljana, Ljubljana, Slovenia

**Keywords:** Gestational diabetes, Illness perception, Attachment styles, Latent profile analysis, Disease burden

## Abstract

**Aims:**

Gestational diabetes (GDM) is a prevalent complication in pregnancy that requires effective self-management, which can be influenced by illness perceptions. Moreover, behavioral regulation can be affected by attachment styles. Thus, our study aimed to identify common GDM perception profiles and test their association with attachment styles.

**Methods:**

In this cross-sectional study, 446 women completed the Relationship Questionnaire (RQ), the Brief Illness Perception Questionnaire (BIPQ), and additional items about GDM diagnosis, information, competence, adherence, behavioral change. Latent profile analysis (LPA) was conducted to determine GDM perception profiles. Multinomial logistic regression followed to calculate the association between GDM perception profiles and attachment styles.

**Results:**

Three distinct profiles emerged: coping (n = 172, 38.6%)—characterized by the most positive GDM perception, burdened (n = 222, 49.8%)—indicating the emotional burden of the disease, and resourceless (n = 52, 11.7%)—reporting lack of resources (i.e. information, competence). Women with insecure attachment styles were more likely to develop a burdened GDM perception profile. Specifically, the expression of a fearful (OR = 1.184 [95%CI: 1.03; 1.36], *p* = 0.016) and a preoccupied (OR = 1.154 [95%CI: 1.01; 1.32], *p* = 0.037) attachment style increased the likelihood for a burdened perception profile, while a secure attachment style (OR = 10.791 [95%CI: 0.65; 0.96], *p* = 0.017) decreased likelihood for developing resourceless GDM perception profile.

**Conclusions:**

Three GDM perception profiles were identified and the role of attachment styles in shaping these perceptions was confirmed. Further studies are needed to investigate whether a tailored treatment approach based on the predominant attachment style could lead to more positive GDM perceptions, improved glycemic control, and better perinatal outcomes.

## Introduction

Gestational diabetes (GDM) is one of the most common complications in pregnancy [1]. Timely detection and good glycemic control are crucial in preventing adverse maternal and neonatal outcomes [2]. However, achieving target glycemic control largely depends on effective self-management. Therefore, it is important to understand the factors influencing an individual’s adherence to treatment recommendations, such as illness perceptions [3].

After a new diagnosis, individuals typically develop their own mental representations of the disease, which may not necessarily align with existing knowledge. The Common-Sense Model of Self-Regulation (CSM) [4] provides a conceptual framework for understanding and examining the perceptual, behavioral, and cognitive processes involved in individuals’ self-management of health threats. Despite this, to the best of our knowledge, only one study in women with GDM has explored illness perception [5], but GDM perceptions have not been studied in detail within the context of CSM. It has been suggested that illness perceptions do not exist in isolation but rather form schemas [4]. Therefore, identifying subgroups of individuals who share similar patterns or profiles of illness perceptions seems to be a reasonable approach. Existing studies in different diseases (i.e. type 2 diabetes [6], cancer [7, 8], rheumatoid arthritis [9], HIV infection [10] have identified two or three profiles including negative, positive, and mixed illness perceptions. However, given the unique challenges of GDM, it is unknown whether similar illness perception profiles are formed. Moreover, with illness perceptions, an individual’s current and future behaviors in coping with the disease can be predicted [11]. Nevertheless, we do not fully understand the etiology of the differences in forming beliefs between individuals. In threatening situations, like in ill-health, the attachment system is activated [12]. Based on Bowlby’s attachment theory [13], Bartholomew and Horowitz [14] proposed four attachment styles: secure, fearful, preoccupied and dismissing. Attachment styles predict many health-related behaviors [15] through at least three pathways: (1) stress regulation, (2) use of external regulators of affect, (3) use of protective factors [16].

Therefore, we hypothesized that in women with GDM, attachment styles may also play a crucial role in the formation of GDM perceptions. We aimed to:

- firstly, identify GDM perception profiles using latent profile analysis (LPA) among newly diagnosed women,
- secondly, assess whether GDM perception profiles are associated with women’s attachment styles.

## Materials and methods

### Study design and setting

A cross-sectional study was conducted at a diabetes outpatient clinic. The GDM diagnosis was made according to the IADPSG criteria [17]. Standard-of-care GDM treatment involved a multidisciplinary lifestyle approach that included group education on diet and exercise, as well as 4-point home blood glucose profile monitoring. The majority of women were treated non-pharmacologically, however, if fasting and postprandial glycemic targets were not achieved at least within 14 days of lifestyle intervention, insulin therapy was initiated.

All questionnaires were applied during the initial appointment at the outpatient diabetes clinic. Women completed the questionnaires in the waiting room while awaiting for a doctor’s appointment. The questionnaires were completed online, using their smartphones. For this purpose, “1 ka” (a Slovenian open-source application enabling an online survey service) was utilized.

The study received approval from the National Medical Ethics Committee of the Republic of Slovenia, case number 0120-449/2021/9.

### Participants

The sample consisted of 446 women with GDM. Women were recruited into the study from July 2022 to December 2022 during their regular appointments at the diabetes outpatient clinic. Inclusion criteria were (1) > 18 years of age, (2) GDM diagnosis, (3) good knowledge of the Slovene language, (4) having agreed to participate. All participating women gave informed consent. The sample’s characteristics can be seen in Table 1.

**Table 1:** Baseline characteristics of the participants according to different attachment styles

	*M* ± *SD* or *f* (%)
Age, years	31.8 ± 5.1
Planned pregnancy	367 (82.3)
Marital status	
Non-marital partnership	243 (54.5)
Married	202 (45.3)
Single	1 (0.2)
Parity	
Nulliparous	219 (49.1)
Multiparous	227 (50.9)
History of GDM	67 (15.0)
BMI, kg/m^2^	26.2 ± 5.7
Education	
Primary education or lower	12 (2.7)
Secondary education	157 (35.2)
BSc or equivalent tertiary education level	99 (22.2)
MSc or equivalent tertiary education level	156 (35.0)
Doctoral degree	22 (4.9)
Gestational age at diagnosis, weeks	19.4 ± 8.3
Gestational age—assessment time, weeks	24.2 ± 8.0

*M *Mean, *SD *Standard deviation, *f *frequency, *GDM* gestational diabetes, *BMI* body mass index

### Measure

#### Sociodemographic data

Data about maternal age, gestational age at the time of the assessment, gestational age at GDM diagnosis, height, weight, parity, GDM in the previous pregnancies, education, employment status, and marital status were gathered with the sociodemographic questionnaire at the study onset.

#### Illness perception

Perceptions about GDM were evaluated using the Brief Illness Perception Questionnaire (BIPQ) [18]. Eight quantitative items evaluating Consequences (Item 1), Timeline (Item 2), Personal control (Item 3), Treatment control (Item 4), Identity (Item 5), Concern (Item 6), Comprehensibility (Item 7), and Emotional response (Item 8) can be used as single item measures or composite. Item 9 is a qualitative, open-ended response item, therefore it was omitted for the purposes of the current study. All of the items are rated using a 0 to 10 response scale, where it is indicated what the two evaluation poles mean for each item. Items 3, 4, and 7 were reversed so that a higher score means more negative perception for all items. The word “illness” was replaced by “gestational diabetes” throughout the questionnaire.

Furthermore, we formed six additional items that tried to capture the main issues that women with GDM highlighted in previously published qualitative studies [19] and based on the experiences of everyday practices with GDM. The participants rated all items on a 0—10 scale. Two evaluation poles were determined for each item.

Items covered (*the evaluation poles are indicated in the parentheses*) (1) the intensity of emotion upon GDM diagnosis (very mild—very intense), (2) hiding the diagnosis (not hiding it at all—hiding it completely), (3) available information about GDM (not enough—enough), (4) competency to follow the GDM recommendations (very little—completely), (5) adherence to the recommendations (not at all—completely), (6) the effort of the lifestyle change (not a bit tiring—very tiring). Additional items 3, 4, 5, and 6 were evaluated in an inverse order, so that higher scores represent more negative perception across all items.

#### Attachment styles

For evaluating the attachment styles, the Relationship Questionnaire (RQ) [14] was used. The RQ is a single-item measure, consisting of four short paragraphs, each describing a prototypical attachment pattern for secure, fearful, preoccupied, and dismissing attachment styles. The questionnaire is divided into two parts; in the first part, participants are asked to select a description under A, B, C, or D paragraph describing each attachment style, that best described them. Hereafter, in the second part, they are asked to rate their agreement with each prototype on a 7-point Likert scale (0—disagree strongly, 7—agree strongly).

### Statistical analysis

Latent profile analysis (LPA) was conducted using R Statistical Software [20] and its package *tidyLPA* [21] to determine the number of latent profiles of participants based on their perception of GDM. To determine the profiles, we used the model where variances for the same construct are constrained to be equal across profiles, and residual covariances are not permitted [22]. An iterative process was implemented, where we started with a single profile and continued to add additional ones until the model no longer improved [22]. The optimal number of profiles was selected based on multiple statistical indices: Log-Likelihood (LL), Akaike information criterion (AIC), Bayesian information criterion (BIC), sample adjusted BIC (SABIC), and bootstrap likelihood ratio test (BLRT). We also took into consideration the principle of parsimony, meaning that each profile had to contain at least 5% of the participants in the sample size to be considered autonomous [23]. Entropy values were also analysed. After selecting the optimal number of profiles, multinomial logistical regression was conducted using IBM SPSS Statistics (Version 27; IBM Corp) to calculate the association between GDM perceptions profile and attachment styles. Multinomial logistic regression was conducted twice, thus producing two separate models (attachment styles as a dichotomous variable or as a continuous variable).

## Results

Among 446 women, only 15% (n = 67) had GDM in a previous pregnancy(-ies) (Table 1).

### Identification of GDM perception profiles

Fit indices for models with an increasing number of profiles are presented in Table 2. We opted for a three-profile solution based on the fit statistics and interpretative consideration of various profile solutions. By adding the number of profiles, all fit indices improved. The largest drop in BIC, AIC, and SABIC was observed for the two-, followed by the four-profile solution, in which also the highest entropy was reached. However, the proportion of the fourth profile in the four-profile solution was small (6.1%), therefore, we decided for the three-profile solution, which also had a sufficient class representation and entropy value.

**Table 2:** Summary of fit statistics for latent profile models based on the illness perceptions

Model	LL	AIC	BIC	SABIC	Entropy	BLRT	*p*(BLRT)	Smallest profile (%)
1 profile	−8852.84	17,761.69	17,875.50	17,787.64				
2 profiles	−8379.53	16,845.07	17,021.38	16,884.92	0.85	946.62	< 0.001	44.8
3 profiles	−8202.80	16,521.61	16,759.43	16,575.36	0.88	353.46	< 0.001	11.7
4 profiles	−7942.48	16,030.96	16,330.28	16,098.61	0.90	520.65	< 0.001	6.1
5 profiles	−7819.87	15,815.74	16,176.57	15,897.30	0.88	245.21	< 0.001	6.1

*LL* log-likelihood, *AIC* Akaike information criterion, *BIC* Bayesian information criterion, *SABIC* sample adjusted BIC, *BLRT* bootstrap likelihood ratio test

**Fig. 1:**
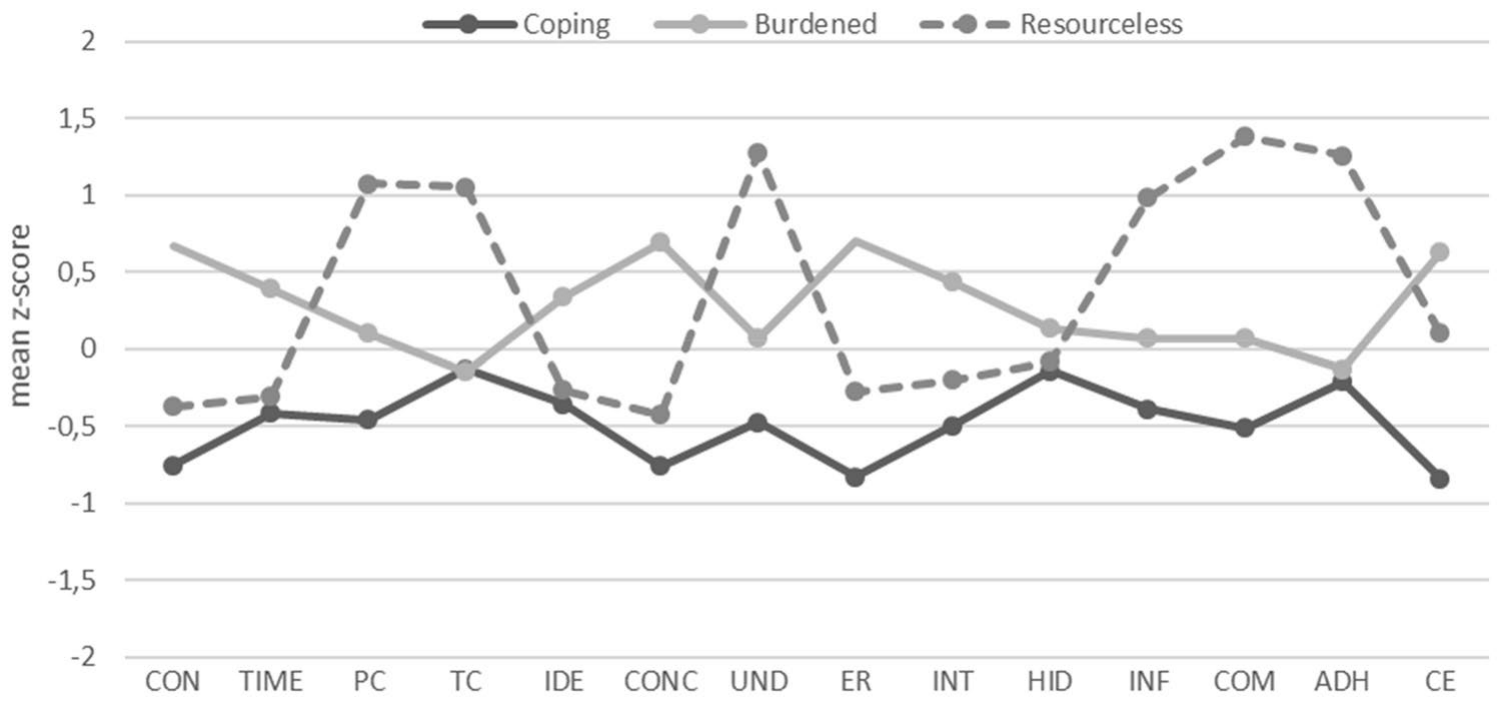
The GDM perceptions profiles characterized by their patterns (mean z-score) of the 14 illness perceptions, where a higher score represents more negative illness perception. Note. CON-Consequences (BIPQ1), TIME-Timeline (BIPQ2), PC-Personal Control (BIPQ3), TC-Treatment Control (BIPQ4), IDE-Identity (BIPQ5), CONC-Concern (BIPQ6), UND-Understanding (BIPQ7), ER-Emotional response (BIPQ8), INT-The intensity of emotions at diagnosis, HID-Hiding the diagnosis, INF-Information, COM-Competence, ADH-Adherence to recommendations, CE-Change effort

The patterns of GDM perceptions that characterized the three GDM perception profiles are illustrated in Fig. 1. Profiles were labeled based on average scores on GDM perception items. The most positive GDM perceptions were observed in the profile labelled *coping*, consisting of 38.6% of participants (n = 172) with the lowest average score on each item, except the item measuring treatment control. The second profile, labelled *burdened*, was the most numerous with 49.8% of participants (n = 222). In contrast to the coping profile, these participants showed the opposite trend, with the most negative rating on items measuring the impact of GDM on their everyday life, experiencing the most GDM symptoms and reporting the highest concern, and emotional response to illness (Table 3). This group also experienced lifestyle changes as the most stressful part of the adjustment to GDM. Lastly, the third profile contained 11.7% of participants (n = 52) and was labelled *resourceless*. Compared to the other two profiles, individuals in this profile experienced the lowest level of personal and treatment control, were lacking information about GDM, had insufficient understanding of the disease, and were the least willing to follow the recommendations.

**Table 3:** GDM perceptions by latent profile (Kruskal–Wallis test with pairwise comparison using Bonferroni correction)

	Whole sample (n = 446)	Coping profile (n = 172)	Burdened profile (n = 222)	Resourceless profile (n = 52)	Profile comparison⁑
	*Mdn* [Q1–Q3]	*Mdn* [Q1–Q3]	*Mdn* [Q1–Q3]	*Mdn* [Q1–Q3]	H
BIPQ1—Consequences	5.0 [3.0–7.0]	3.0 [1.0–4.0]	7.0 [5.0–8.0]	4.0 [2.0–5.0]	212.07*** 1 < 2 < 3
BIPQ2—Timeline	2.0 [0.0–4.0]	0.0 [0.0–2.5]	3.0 [2.0–5.0]	1.5 [0.0–2.5]	78.45*** 1 < 2; 2 > 3; 1 = 3
BIPQ3—Personal control	2.0 [1.0–4.0]	1.0 [0.0–2.5]	3.0 [2.0–4.0]	5.0 [3.5–6.0]	97.34*** 1 < 2 < 3
BIPQ4—Treatment control	2.0 [0.0–5.0]	2.0 [0.0–5.0]	2.0 [1.0–4.0]	5.5 [5.0–7.0]	59.51*** 1 = 2, 1 < 3; 2 < 3
BIPQ5—Identity	1.0 [0.0–3.0]	0.0 [0.0–2.0]	2.0 [0.0–5.0]	1.0 [0.0–2.0]	45.16*** 1 < 2; 1 = 3; 2 > 3
BIPQ6—Concern	4.0 [2.0–6.0]	2.0 [0.0–3.0]	6.0 [5.0–7.0]	2.0 [1.0–5.0]	222.00*** 1 < 3; 2 > 3; 1 = 3
BIPQ7—Understanding	2.0 [1.0–5.0]	1.0 [0.0–3.0]	3.0 [2.0–5.0]	5.0 [5.0–7.0]	107.28*** 1 < 2 < 3
BIPQ8—Emotional response	5.0 [2.0–7.0]	2.0 [0.0–3.0]	7.0 [5.0–8.0]	4.0 [2.0–5.0]	237.39*** 1 < 2; 1 < 3; 2 > 3
AdI1—The intensity of emotion at diagnosis	5.0 [3.0–7.0]	3.0 [1.5–5.0]	6.0 [5.0–8.0]	5.0 [3.0–5.0]	86.70*** 1 < 2; 1 = 3; 2 > 3
AdI2—Hiding the diagnosis	0.0 [0.00–0.0]	0.0 [0.0–0.0]	0.0 [0.0–0.0]	0.0 [0.0–0.0]	8.77* 1 > 2; 1 = 3; 2 = 3
AdI3—Information	2.0 [0.0–4.0]	1.0 [0.0–3.0]	2.0 [1.0–4.0]	5.0 [3.0–7.0]	72.78*** 1 < 2 < 3
AdI4—Competence	2.0 [0.0–3.0]	0.0 [0.0–2.0]	2.0 [1.0–3.0]	5.0 [3.5–6.5]	122.69*** 1 < 2 < 3
AdI5—Adherence to the recommendation	1.0 [0.0–2.0]	1.0 [0.0–2.0]	1.0 [0.0–2.0]	3.0 [2.0–5.0]	66.83*** 1 = 2; 2 < 3; 1 < 3
AdI6—Change effort	5.0 [3.0–7.0]	2.0 [1.0–4.0]	7.0 [6.0–8.0]	5.0 [3.5–7.0]	212.06*** 1 < 2; 2 > 3; 1 < 3

*AdI* Additional item

⁑ Results of the Kruskal–Wallis test comparing profile groups, coded as follows: 1 = coping, 2 = burdened, 3 = resourceless

*p < 0.05, **p < 0.01, ***p < 0.001

Next, we explored whether GDM perception profiles were associated with women’s attachment styles. Of the participants, 324 (72.6%) recognized the secure attachment style as their predominant pattern, while the remaining 123 (27.4%) selected one of the insecure attachment styles: 68 (15.2%) fearful attachment style, 32 (7.2%) preoccupied attachment style, and 22 (4.9%) dismissing attachment style. No differences in baseline characteristics were observed between groups of women with different attachment styles.

In the multinomial logistic regression, *coping* profile was chosen as the reference. In model 1, sociodemographic characteristics and dichotomous attachment style variables (distinguishing between women with secure and insecure attachment styles) were included as independent variables. Higher education was statistically significantly associated with burdened group classification. Additionally, lower maternal age was associated with resourceless group classification (Table 4). Holding sociodemographic variables constant, women with an insecure attachment style were 44.7% more likely to be classified into the burdened GDM perception profile than women with a secure attachment style.

**Table 4:** Results of multinomial logistic regression models predicting burdened or resourceless GDM perception profile in comparison with coping profile (n = 446)

	Burdened profile (n = 222)				Resourceless profile (n = 52)			
	*B*	OR	95% CI	*p*	*B*	OR	95% CI	*p*
*Model 1*								
Intercept	−0.841				−0.421			
Age	−0.008	0.992	[0.95; 1.04]	0.716	−0.080	0.923	[0.86; 0.99]	**0.027**
BMI	0.033	1.04	[1.0; 1.07]	0.086	0.039	1.04	[0.98; 1.10]	0.173
Education	0.203	1.23	[1.02; 1.45]	**0.032**	−0.030	0.971	[0.74; 1.28]	0.835
History of GDM	0.259	1.30	[0.73; 2.3]	0.377	0.429	1.536	[0.58; 4.08]	0.389
Gestational age—diagnosis	−0.021	0.979	[0.96; 1.00]	0.102	0.022	1.02	[0.98; 1.06]	0.278
Attachment style (Ref-insecure)	−0.805	0.447	[0.28; 0.73]	**0.001**	−0.577	0.562	[0.27; 1.16]	0.117
*Model 2*								
Intercept	−1.916				−0.078			
Age	−0.001	0.999	[0.96; 1.04]	0.965	−0.083	0.920	[0.86; 0.99]	**0.023**
BMI	0.035	1.036	[1.00; 1.08]	0.072	0.043	1.044	[0.99; 1.11]	0.144
Education	0.206	1.229	[1.02; 1.49]	0.034	0.030	1.030	[0.77; 1.37]	0.839
History of GDM	0.208	1.231	[0.68; 2.22]	0.489	0.508	1.662	[0.61; 4.54]	0.322
Gestational age—diagnosis	−0.023	0.978	[0.95; 1.00]	0.084	0.022	1.023	[0.98; 1.07]	0.280
Attachment styles								
Secure	−0.082	0.922	[0.81; 1.05]	0.231	−0.235	0.791	[0.65; 0.96]	**0.017**
Fearful	0.169	1.184	[1.03; 1.36]	**0.016**	−0.037	0.964	[0.78; 1.20]	0.740
Preoccupied	0.143	1.154	[1.01; 1.32]	**0.037**	0.175	1.192	[0.98; 1.45]	0.079
Dismissing	−0.019	0.981	[0.87; 1.11]	0.761	−0.114	0.892	[0.73; 1.09]	0.255

*BMI* body mass index, *GDM* gestational diabetes

In model 2 (Table 4) the expression of each attachment style was used as a continuous independent predictor. Lower age was significantly associated with a resourceless profile. Controlling for covariates, the odds of developing a burdened GDM perception profile increased by 18.4% if the expression of fearful or preoccupied attachment style increased by one, respectively. On the other hand, the odds ratio to develop a resourceless perception profile was 20.9% lower if the expressiveness of the secure attachment style increased by one.

## Discussion

Our study with a person-centred approach provided the first data on GDM perception profiles among newly diagnosed women. We demonstrated that women with GDM differed in their perceptions, revealing the existence of three distinct GDM perception profiles. Furthermore, the findings support the role of attachment styles in shaping different GDM perception profiles. Specifically, women with predominantly insecure attachment patterns were more likely to develop a more burdensome perception of GDM. Conversely, women with a secure attachment style were less likely to be assigned to the resourcesless GDM perception profile.

To the best of our knowledge, no study has focused on studying GDM perception profiles, nor have they examined the differences in the formation of illness perception according to different attachment styles. Three illness perception profiles (coping, burdened, and resourceless) were identified, aligning with previous studies where the number of profiles ranged from two to three, typically including at least one positive and one negative illness perception profile [6, 8, 24]. However, studies across different illnesses with three identified profiles often showed that the third profile unites individuals with moderate illness perception [10, 24], which was not found in our study. Apart from the coping profile, characterized by the most positive GDM perceptions, two groups with more negative GDM perceptions were identified. The burdened group comprised women who experienced the most symptoms (even though GDM is an asymptomatic disease), were the most concerned, experienced the highest impact of the disease on their everyday life, and experienced the lifestyle change needed as the most difficult. The recourceless group encompassed individuals with significantly fewer resources, such as personal or treatment control, information, and a lower willingness to adhere to recommendations. The distinction between these two groups with more negative perceptions seemed very clear: the first group experienced a higher (emotional) burden of the disease, while the second group felt less empowered, without reporting significant distress.

The second aim of our study was to assess if the GDM perception profiles are associated with women’s attachment style. Women with insecure attachment styles were more likely to develop a burdened profile. Additionally, the burdened profile was more likely to be developed by individuals with higher expressions of fearful or preoccupied attachment style. On the other hand, a higher expression of a secure attachment style was negatively associated with assignment to a resourceless GDM perception profile. Our results align with existing knowledge, where anxious-ambivalent individuals tend to be overly vigilant to negative events or stressful situations, reacting with strong emotional distress [12]. Interestingly, the expressiveness of dismissing attachment style, associated worse glycemic control and adherence in type 1 and 2 diabetes [25], was not significantly associated with GDM perception profiles in our study. Maunder and Hunter [26] explained that dismissingly attached individuals are more prone to minimizing their symptoms. It can be that these individuals also underreported their beliefs about GDM. In the future, tailoring communication to each attachment style could be useful, investigating whether it helps develop more realistic perceptions about GDM and potentially adjusting pathways of care around different perception profiles.

Among sociodemographic characteristics, the likelihood of having a burden profile of GDM perceptions increased with increasing education, while the likelihood of a resourceless profile increased with decreasing age, both compared with the coping profile. The positive relationship between education level and maternal health [27], and well as better glycemic control in diabetes [28], is well documented. However, some studies also showed that more educated women can be prone to higher psychological distress [29], which may partly explain our results. Furthermore, women with higher levels of education are more likely to have a healthy lifestyle, including a healthy diet [30], which is the cornerstone of GDM treatment. In line with that, they probably had fewer opportunities for lifestyle improvement, having taken care of their health before pregnancy. This may explain experiencing higher distress when diagnosed with GDM and a higher burden of lifestyle change needed. Moreover, they might have felt helplessness because they received the diagnosis despite taking care of their health. On the other hand, it was shown that younger women were more likely to have a resourceless GDM perception profile with less information and feeling less in control of taking care of GDM. In some studies, it was shown that younger women had lower GDM knowledge assessed with a test [31], but others did not confirm that [32]. Considering our results, we could not distinguish if younger women objectively had less knowledge or if they just felt less competent. Therefore, it would be appropriate to further identify and address the barriers that prevent these women from feeling empowered and competent following current uniform education.

To the best of our knowledge, this is the first study examining GDM perceptions profiles. Moreover, it is also the first study assessing the associations between attachment style and illness perception profiles. However, the limitations must be considered. The main limitation is associated with the cross-sectional design, which does not allow us to draw causal conclusions. The predominantly well-educated, Caucasian sample of women limits the generalizability. The results are also limited by the nature of the BIPQ, which measure different illness perception with only one item. Therefore, GDM-specific items have been added, however, those were not validated before. In this regard, our results should be validated also in other populations. Nevertheless, LPA is a powerful statistical procedure, but it does not guarantee the correct assignment of respondents to classes. Future studies should also include the glucose control data and perinatal data to evaluate whether differences in attachment styles influence GDM clinical outcomes.

In summary, three distinct GDM perception profiles were identified. Furthermore, we demonstrated that insecure attachment styles were significantly associated with assignment to the burdened GDM perception profile, indicative of a high emotional burden of the disease in everyday life. Characteristics specific to each GDM perception profile, considering attachment style and sociodemographic characteristics, could be used to target information in routine patient communication.
